# Editorial: Gene Targeting in Neuroscience: Entering the Future

**DOI:** 10.3389/fgene.2016.00077

**Published:** 2016-05-06

**Authors:** Robert Gerlai

**Affiliations:** Department of Psychology, University of Toronto MississaugaMississauga, ON, Canada

**Keywords:** gene targeting techniques, CRISPR/Cas9, TALEN, brain, DREADDs, CRE recombinase

Many methods are available for the manipulation of brain function and behavior. From pharmacological tools through electrophysiological techniques to neuroanatomical procedures, an arsenal of approaches has been successfully utilized to answer the question of how the brain works and what may be behind its dysfunction in human disorders. Why do many scientists prefer to use genetics then? There may be two principally different reasons for this choice: one relates to evolution, the other to practical or mechanistic points. Ultimately, all behavioral features of an organism, and thus all functional aspects of its brain, must pass the test of natural selection. Natural selection operates at the level of genes. In other words, albeit often complex and difficult to disentangle, the effects of genes on brain function and behavior is not questionable. The second reason is more practical, and concerns how efficiently a biologist can answer mechanistic questions. The cause of human CNS disorders is often multifaceted, and is usually expected to involve numerous unknown environmental factors. Simply put, the number of such environmental factors could be staggering, whereas the number of genes one can manipulate is only about 25,000. Identifying a gene is also much simpler than identifying environmental agents possibly involved in brain dysfunction. A good example for this reasoning is the discovery of the involvement of Amyloid Precursor Protein (APP) misprocessing in Alzheimer's Disease. Only <5% of Alzheimer cases are familial, i.e., heritable, yet it was the discovery, for example, of APP mutations and the mutations in APP processing enzymes that propelled the understanding of the disease forward. Another mechanistic reason why genetic manipulation is often preferred to other methods is the specificity of the former. Modern recombinant DNA methods allow one to precisely silence a single gene, or change its expression in a brain region specific manner, or even modulate neuronal function in particular circuits and at particular time points. This level of control, temporal and spatial resolution, as well as target specificity are often not achievable with methods other than genetics. The current special topic will present numerous such genetic techniques, the first, and perhaps the most famous of which being homologous recombination-based gene targeting in embryonic stem cells.

The double selection scheme that allowed efficient identification of homologous recombination-based replacements of endogenous genes with a targeting vector in embryonic stem cells has revolutionized reverse genetics. The method, developed by Mario Capecchi and his colleagues in the mid-80s, which became known as gene targeting or the knock out technology, has since been widely used in studies investigating the function of genes in practically every sub-discipline of biology. This special topic presents a collection of papers that focus on how gene targeting has been utilized in neurobiology, i.e., in the analysis of the biological mechanisms of brain function and behavior. It reviews classical (first generation) knock out methods and second generation (inducible and cell type restricted gene expression and targeting) techniques, including the CRE-recombinase system, the evolution of the technology as well as the evolution of its use (Tsien; Gerlai). The latter papers describe what we have learned over the past three decades about the utility and the limitations of gene targeting in brain research, and what the potential future of this technology may be. The special topic also extends the question of how gene targeting is employed for the discovery of gene function to how it may be used to generate animal models of human brain disorders (Leung and Jia). In the past decade, novel genome engineering tools have also been developed. These new methods now compete with the true and tried ES cell based gene-targeting technique. For example, the Transcription activator-like effector nuclease (TALEN) system now allows one to custom design restriction endonuclease-like enzymes that will cut specifically at the desired nucleotide sequence (Lee et al.). Similarly, the Clustered regularly-interspaced short palindromic repeats (CRISPR) and CRISPR associated protein 9 (Cas9) system now also enables the researcher to create double stranded cuts at pre-specified nucleotide sequences of choice (Walters et al.). Importantly, both the TALEN and the CRISPR/Cas9 system can be employed in species other than the mouse or the rat, the two laboratory model organisms to which ES cell based gene-targeting methods of the past were restricted. Last, the special topic also reviews the Designer Receptors Exclusively Activated by Designer Drugs (DREADD) technology (Whissell et al.). Although not a gene targeting method per se, DREADD is a recombinant DNA manipulation-based approach that combines genetics with pharmacology and allows one to specifically target particular neuronal populations and conduct a high temporal resolution analysis of their function.

As the saying goes, “clearly we are living exciting times” (unconfirmed source, but believed to originate from a Chinese curse). The investigator now has an array of modern recombinant DNA-based tools with which he or she can manipulate brain function and/or behavior. The number of such tools is continually growing, and the context in which they are employed is increasing in breadth. This special topic only captures a fraction of these methods and the different ways they may be employed. Nevertheless, it attempts to paint an objective picture discussing the pros and cons of these tools. As such, it may be a useful read for the novice neurobehavioral geneticist and the expert neurobiologist alike. It may be of value to scientists of other fields of biology as well who wish to employ these techniques for purposes other than the investigation of the brain. Last, we feel the papers may be useful for both instructors and students who are interested in the rapidly evolving field of neurobehavioral genetics and modern behavioral neuroscience.

## Author contributions

The author confirms being the sole contributor of this work and approved it for publication.

### Conflict of interest statement

The author declares that the research was conducted in the absence of any commercial or financial relationships that could be construed as a potential conflict of interest.

